# Inflammation and Oxidative Stress in Diabetic Nephropathy: New Insights on Its Inhibition as New Therapeutic Targets

**DOI:** 10.1155/2013/248563

**Published:** 2013-06-03

**Authors:** Akira Mima

**Affiliations:** Department of Nephrology, Graduate School of Medicine, Institute of Health Biosciences, University of Tokushima, Tokushima 770-8503, Japan

## Abstract

Diabetes and insulin resistance can greatly increase microvascular complications of diabetes including diabetic nephropathy (DN). Hyperglycemic control in diabetes is key to preventing the development and progression of DN. However, it is clinically very difficult to achieve normal glucose control in individual diabetic patients. Many factors are known to contribute to the development of DN. These include diet, age, lifestyle, or obesity. Further, inflammatory- or oxidative-stress-induced basis for DN has been gaining interest. Although anti-inflammatory or antioxidant drugs can show benefits in rodent models of DN, negative evidence from large clinical studies indicates that more effective anti-inflammatory and antioxidant drugs need to be studied to clear this question. In addition, our recent report showed that potential endogenous protective factors could decrease inflammation and oxidative stress, showing great promise for the treatment of DN.

## 1. Introduction

Diabetes nephropathy (DN) is the major determinant of morbidity and mortality in patients with diabetes. Chronic hyperglycemia is a major initiator of DN. Several studies indicate a causal link between the degree of glycemic control in patients with diabetes and the development and progression of complications. The Diabetes Control and Complications Trial (DCCT) demonstrated that intensive glycemic control in patients with both type 1 and 2 diabetes successfully delayed the onset and retarded macrovascular and microvascular complications including DN [1, 2]. In addition, the United Kingdom Prospective Diabetes Study (UKPDS) indicated that intensive glycemic control in patients with type 2 diabetes decreased the risk of DN and diabetic retinopathy [3, 4]. Thus, strict glycemic control could prevent the initiation and development of DN. Despite such lines of evidences conventional therapies used for glycemic control in patients with diabetes do not always prevent the ultimate progression of DN. Therefore, the use of therapies that specifically target DN could be useful and really needed in addition to a strict glycemic control. It is reported that the induction of inflammation and oxidative stress by the metabolism of hyperglycemia and dyslipidemia may play a significant role in developing vascular complications including DN in patients or animals [5–9]. The increases of inflammatory cytokines and reactive oxygen species (ROS) have also been shown in DN. Our recent studies clearly showed that insulin or glucagon-like peptide-1 (GLP-1) prevented the development of DN, neutralizing inflammation and oxidative stress [10]. This paper will outline these theories and the potential therapeutic interventions that could prevent DN in the presence of hyperglycemia and dyslipidemia.

## 2. Induction of Inflammation by Type 1 and Type 2 Diabetes

Type 1 diabetes is characterized by a progressive cell-mediated destruction of pancreatic islet cells, leading to loss of insulin production. The development of DN is associated with significant inflammatory cells infiltration with increasing in plasma levels of CRP and inflammatory cytokines such as vascular cell adhesion molecule-1 (VCAM-1) and interleukin (IL)-1*β* [11]. These data strongly support that immune cells participate in the development of DN. 

Increases in inflammation are detected in healthy individuals who later go on to develop type 2 diabetes; several reports indicate that, in type 2 diabetes, insulin resistance, CD8^+^ T cells are activated in obese adipose tissue [12]. Further, it is reported that healthy, middle-aged women who showed high levels of inflammatory markers IL-6 and CRP had increased risk for developing type 2 diabetes over a 4-year period [13]. 

## 3. Induction of Oxidants by Type 1 and Type 2 Diabetes

There are several studies showing that oxidant production is increased in both type 1 and type 2 diabetes. Oxidative stress results when the rate of oxidant production exceeds the rate of oxidant scavengers and also by alteration of nicotinamide adenine dinucleotide phosphate (NADPH)/NADP ratios results [14]. The abnormal metabolism of glucose or free fatty acid (FFA) via mitochondria pathways and the activation of NADPH oxidases via protein kinase C (PKC) have been recognized as contribution of oxidant production [15]. There is substantial evidence supporting that ROS is increased in kidney and retina either exposed to hyperglycemia or in diabetic animals [16–18]. Further, plasma levels of 8-hydroxydeoxyguanosine, isoprostanes, and lipid peroxides are elevated both in diabetic animals and patients [19–21]. Thus, increased ROS production in diabetes can originate from the abnormal metabolism of glucose and FFA through multiple pathways. This supports an explanation for the findings of increased oxidative stress in insulin-resistant nondiabetic patients.

## 4. Association between Inflammatory Processes and Diabetic Nephropathy

Although metabolic and hemodynamic factors are the main causes of DN, recent studies have suggested that DN is an inflammatory process, and immune cells could be involved in the development of DN [22, 23]. Hyperglycemia may induce macrophage production of IL-12, which can stimulate CD4 cell production of IFN-*γ*. FFA, hyperglycemia, and obesity may activate nuclear factor *κ*B (NF-*κ*B) through PKC and ROS to rapidly stimulate the expression of cytokines [24, 25]. Following activation, NF-*κ*B translocates to the nucleus, stimulating rapidly the subsequent transcription of genes such as endothelin-1 (ET-1), VCAM-1, intercellular adhesion molecule-1 (ICAM-1), IL-6, and TNF-*α* that promote the development of DN. It is well known that elevated levels of advanced glycation end products (AGE) can be found in kidney. AGE interacts with receptor for AGE (RAGE), and AGE/RAGE interactions have been reported in the development of DN [26]. Exposure of activated lymphocytes to AGE enhances the expression of IFN-*γ* which may accelerate immune responses that contribute to developing DN [27, 28]. Additionally, in a clinical study of type 2 diabetes patients, positive correlations were recognized between plasma IFN-*γ*, proteinuria, and estimated glomerular filtration rate (eGFR) [29]. Further, plasma IL-2R levels found in type 2 DM patients with overt DN were higher than in those without overt nephropathy, and apparent positive correlation was recognized between plasma IL-2R and proteinuria [29]. 

## 5. The Role of Oxidative Stress in Diabetic Nephropathy

Numerous studies clearly indicate that both diabetic state and insulin resistance play a central role in producing oxidative stress; free glucose activates aldose reductase activity and the polyol pathway, which decreases NADPH/NADP^+^ ratios [30]. Elevated intracellular glucose activates PKC through de novo synthesis of diacylglycerol (DAG) [31]. Activation of PKC in the glomeruli has been associated with processes increasing mesangial expansion, thickening basement membrane, endothelial dysfunction, smooth muscle cell contraction, and activation of cytokines and transforming growth factor-*β* (TGF-*β*) [32]. PKC induces oxidative stress by activating mitochondrial NADPH oxidase [14]. Vascular NADPH oxidase consists of multiple subunits including phox47, phox67, and Nox isoforms [14]. ROS generated from Nox isoforms might induce endothelial dysfunction, inflammation, and apoptosis [33]. Excess FFA, mainly derived from insulin-resistant state, also can increase oxidant production by *β* oxidative phosphorylation via mitochondrial metabolism [34, 35]. Studies using rodents indicate that increases in oxidative stress could be responsible for developing DN; inhibition of the polyol pathway with aldose reductase inhibitors could reduce the effects of hyperglycemia on DN [36]. Further, administration of vitamin C or E has been shown to be effective in ameliorating rodent model of DN [37, 38]. Another study has also shown that high doses of vitamin E normalized parameters of oxidative stress and inhibited vascular abnormalities caused by DAG-PKC activation in the kidney [39].

## 6. Antioxidants as Therapeutics for Diabetic Nephropathy

### 6.1. Vitamin C and E

Administration of vitamin C, alone or in combination with vitamin E, has suggested decreases in microalbuminuria. Patients with type 1 diabetes for less than 10-year duration who received high doses (1,800 IU/day) of vitamin E showed restoration of renal function [37]. However, these studies were of very short duration and small sample size. In contrast, in the 4-year-long Heart Outcomes Prevention Evaluation (HOPE) study of more than 3,600 diabetic patients, some of whom already displayed microalbuminuria, vitamin E supplementation (400 IU/day) did not lower the risk for cardiovascular outcome significantly [40]. Thus, based on these lines of evidence, effectiveness using vitamin C or E toward DN remains to be unknown.

### 6.2. Nrf2

The transcription factor NFE2-related factor 2 (Nrf2) is a master regulator of cellular detoxification responses and redox status. Upregulation of Nrf2 and its downstream antioxidant genes in response to hyperglycemia was found both in the kidney and renal cells [41–43]. In clinical trials reported recently, bardoxolone methyl, which interacts with cysteine residues on Keap1, allowing Nrf2 translocation to the nucleus leading to anti-inflammatory effects, appears to have beneficial effects in DN compared with placebo after 52 weeks of treatments [44, 45]. However, this phase 3 trial was halted in October 2012 because of a higher mortality in treated group.

## 7. Anti-Inflammatory Drugs as Therapeutics for Diabetic Nephropathy

 Inflammation plays a significant role in developing DN in several models of DN; DN reflects the results of inflammatory, metabolic, and hemodynamic factors. Inflammation could cause glomerulosclerosis, tubular atrophy, and fibrosis. Therefore, administration of anti-inflammatory strategies could be a potential treatment of DN. 

### 7.1. MCP-1 and CCL2 Inhibitor

 Monocyte-chemoattractant protein-1 (MCP-1) is significantly increased in DN, and macrophage infiltration into glomeruli is associated with glomerular injury. Further, urinary excretion of MCP-1 is correlated with diabetic glomerular injury [5]. MCP-1 null mice are protected against DN [46] and blockade of the MCP-1 receptor, C-C chemokine receptor type 2 (CCR-2) using propagermanium-ameliorated diabetic glomerulosclerosis [47]. However, clinical inhibitors of chemokine C-C motif ligand 2 (CCL2) inhibitor may show partial effects to DN [48], because even complete deletion of CCL2 only reduced albuminuria in rodent DN model [49].

### 7.2. Pentoxifylline

Tumor necrosis factor-*α* (TNF-*α*) is mainly produced by monocytes and macrophages. However, TNF-*α* expression is increased in the kidney in DN. It has been reported that patients with type 2 diabetes have 3-4 times greater serum levels of TNF-*α*, compares to healthy control [50]. Further, the level of TNF-*α* was higher in DN with microalbuminuria compared within those with no albuminuria [51]. Pentoxifylline inhibits the expression of mRNA levels of TNF-*α* [52]. Combination with angiotensin-converting enzyme inhibitors (ACEI), AT1 receptor blockers (ARB), and pentoxifylline could decrease albuminuria in DN [53, 54].

### 7.3. Adipokines

 Adiponectin derived from adipose tissue has anti-inflammatory properties. Adiponectin suppresses inflammatory markers including TNF-*α*, receptor activation for platelet-derived growth factor (PDGF), epidermal growth factor (EGF) or fibroblast growth factor (FGF) [55]. Diabetic rat overexpression of adiponectin might preserve nephrin, decrease expression levels of TGF-*β*, and reduce albuminuria [56]. However, it is still unclear whether adiponectin will provide significant effects toward human DN.

### 7.4. Inhibition of NF-*κ*B

 Recent studies suggest the critical role of NF-*κ*B in the development of insulin resistance, including an animal model [57] and a human study [58]. Salsalate treatment improved insulin-sensitivity-increased adiponectin, resulting in reduction of inflammatory markers [58]. For DN, several lines of evidence support the pivotal role of NF-*κ*B in the development of DN including mesangial cells [59], glomerular endothelial cells [10], and podocytes [60]. Inhibition of NF-*κ*B in kidney using peroxisome proliferator-activated receptor-*γ* (PPAR-*γ*) [61], ARB [62], or pentosan polysulfate (PPS) [63] may ameliorate DN in animal model. However, clear demonstration of the efficacy of inhibition of NF-*κ*B in delaying progression of DN has not been reported.

### 7.5. HMG-CoA Reductase Inhibitors

3-Hydroxy-3-methyllutaryl CoA (HMG-CoA) reductase inhibitors, or statins, are potent inhibitors of cholesterol biosynthesis that are useful for treatment of patients with dislipidemia. HMG-CoA reductase inhibitors might prove to be key inhibitors of low-grade inflammation and endothelial dysfunction by reducing inflammatory cell signaling [64]. In clinical studies, the beneficial effect of statins on renal function in DN is still controversial; in a subanalysis of the Treating to New Targets (TNT) study, treatment with 10 mg and 80 mg atorvastatin was found to increase eGFR [65], while in the Prevention of Renal and Vascular End-Stage Disease Intervention Trial (PREVEND-IT), treatment with 40 mg pravastatin did not result in increases in eGFR [66]. 

### 7.6. Rapamycin

 The mammalian target of rapamycin (mTOR) is a serine/threonine kinase that mediates cell proliferation, survival, size, and mass [67]. Rapamycin reduces mTOR activity that is increased by hyperglycemia and mediates the renal changes in DN, including mesangial expansion or glomerular basement thickness [68]. Rapamycin significantly reduces the influx of inflammatory cells including monocytes and macrophages associated with progression of DN [69, 70]. Rapamycin also reduces the release of proinflammatory cytokines or chemokines including MCP-1, regulated and normal T cell expressed and secreted (RANTES), IL-8, and fractalkine [69, 70]. Thus, administration of rapamycin as an anti-inflammatory drug could be a new therapeutic regimen for DN.

### 7.7. Aspirin and COX-2 Inhibitors

Aspirin and cyclooxygense-2 (COX-2) inhibitors are major anti-inflammatory agents. Recent study has suggested that administration of aspirin could decrease albuminuria in patients with DN [71]. Further, combination with aspirin and AT1 receptor blockers (ARB) resulted in a further decrease in the progression of DN and inflammatory markers compared to aspirin treatment alone [72]. It is reported that COX-2 inhibitors could increase renal hemodynamics and decrease profibrotic cytokines [73]. Despite this report, treatment with 200 mg/day COX-2 inhibitor for six weeks could not decrease DN [74]. Thus, administration of COX-2 inhibitors for treatment of DN remains controversial. 

### 7.8. Inhibition of PKC Activation

 As described previously, the activation of PKC is induced by hyperglycemia and insulin resistance. It has been reported that PKC activation altered cell signaling molecules including inflammatory cytokines such as NF-*κ*B, IL-6, TNF-*α*, and plasminogen activator-1 (PAI-1) in many vascular cells including endothelial cells and mesangial cells [10, 75, 76]. Ruboxistaurin (RBX), a PKC*β* isoform selective inhibitor, has been shown to prevent DN in rodent DN models through inhibition of mediators of extracellular matrix accumulation, TGF-*β* and amelioration of insulin signaling [77, 78]. Further, diabetic PKC*β* null mice showed decreases of albuminuria and mesangial expansion [79]. In a phase II clinical trial it was shown that RBX treatment with diabetes significantly decreased albuminuria and maintained a stable estimated glomerular filtration rate (eGFR) [80]. Recently, we showed that hyperglycemia can activate PKC*β* isoforms, which enhance angiotensin II (Ang II) toxic effect in glomerular endothelial cells and decrease glucagon-like peptide-1 (GLP-1) receptor, leading to resistance of GLP-1's treatment on DN [10]. 

 Further, our recent findings suggest that hyperglycemia activates PKC*δ* and p38 mitogen-activated protein (MAPK) to increase Src homology-2 domain-containing phosphatase-1 (SHP-1) and causes VEGF resistance and independent NF-*κ*B activation to induce podocyte apoptosis in DN [60].

### 7.9. Insulin and NO

 It is reported that exogenous insulin could inhibit the activation of TNF-*α* in animal models [81]. Also, other reports suggested that insulin could inhibit MCP-1 expression and activation of NF-*κ*B in endothelial cells [82]. Furthermore, recent studies in patients with type 2 diabetes showed that insulin treatment decreased expressions of inflammatory cytokines, such as MCP-1, ICAM-1, soluble VCAM-1 (sVCAM-1), TNF-*α*, and IL-6 [83, 84]. 

 Insulin can increase endothelial nitric oxide (NO) production by rapid posttranslational mechanisms, which are mediated by the PI3K/Akt signaling pathway, resulting in vasodilatation, antithrombotic effect, and anti-inflammation [78, 85, 86]. Insulin stimulates not only NO production but also the expression of endothelial NO synthase (eNOS) in endothelial cells [87]. Supporting this, a recent study indicates that vascular endothelial cell specific insulin receptor knockout mice decreased eNOS expression in aorta [88]. Thus, impairment of insulin action in vascular tissue could contribute to DN. However, the efficacy of exogenous NO donor remains unclear.

### 7.10. PPAR-*γ* Agonist

Peroxisome proliferator-activated receptors (PPARs) have been shown to have pivotal role in regulating insulin sensitivity, lipid metabolism, adipogenesis, and cell growth [89–92]. Recent studies indicated that PPAR-*γ* agonist decreased expressions of inflammatory markers such as PAI-1, ICAM-1, and NF-*κ*B in the kidney in DN and ameliorated renal function [93]. 

### 7.11. GLP-1 and DPP-IV Inhibitors

 The incretin hormone, GLP-1, is released from gut in response to meal which can augment glucose-dependent insulin release [94]. Analysis of GLP-1 receptor (GLP-1R) revealed its expression in endothelial cells and kidney [10, 95, 96]. In endothelial cells, GLP-1 might inhibit the expression of TNF-*α* and VCAM-1 [10]. Our recent study indicates that GLP-1 is partly mediating its protective actions via its own receptor by the activation of protein kinase A (PKA) [10]. GLP-1 can induce protective actions on the glomerular endothelial cells by diminishing the signaling pathway of Ang II at phospho-c-Raf(Ser338)/phospho-Erk1/2 via phospho-c-Raf(Ser259) activated by cAMP/PKA pathway. Further, administration of GLP-1 in DN decreased inflammatory markers including PAI-1, CD68, IL-6, TNF-*α*, NF-*κ*B, and CXCL2 in the kidney [10]. Thus, our recent studies indicated the mechanisms by which GLP-1 could induce protective actions on the glomerular endothelial cells by inhibiting the signaling pathway of Ang II and its proinflammatory effect, and indicated a dual signaling mechanism by which PKC*β* activation could increase Ang II action and inhibit GLP-1's protective effects by reducing the expression of GLP-1 receptors in the glomerular endothelial cells.

**Figure 1:**
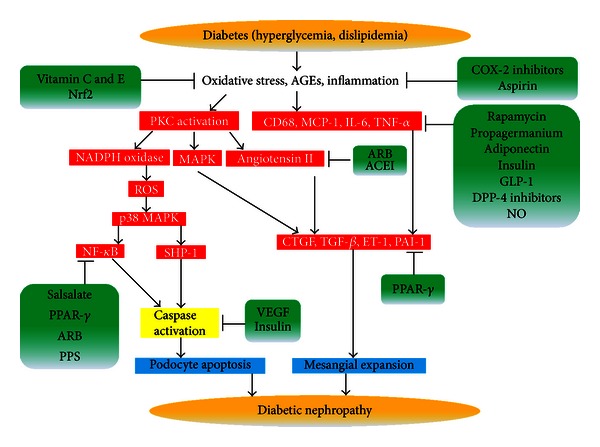
Schematic diagram on the progression of diabetic nephropathy and its inhibition. AGEs: advanced glycation end products; PKC: protein kinase C; COX-2: cyclooxygense-2; Nrf2: NFE2-related factor 2; NADPH: nicotinamide adenine dinucleotide phosphate; MAPK: mitogen-activated protein kinase; MCP-1: monocyte-chemoattractant protein-1; IL-6: interleukin-6; TNF-*α*: tumor necrosis factor-*α*; GLP-1: glucagon like peptide-1; DPP-4: dipeptidyl peptidase-4; NO: nitric oxide; ARB: AT1 receptor blockers; ACEI: angiotensin-converting enzyme inhibitors; NF-*κ*B: nuclear factor-*κ*B; CTGF: connective tissue growth factor; TGF-*β*: transforming growth factor-*β*; VEGF: vascular endothelial growth factor; SHP-1: Src homology-2 domain-containing phosphatase-1; PPAR-*γ*: peroxisome proliferator-activated receptor-*γ*; PPS: pentosan polysulfate.

Dipeptidyl peptidase-4 (DPP-4) inhibitors provide vascular protection by increasing GLP-1's bioavailability and its action. It has been reported that DPP-4 inhibitors reduced MCP-1. Further, DPP-4 inhibitors have vasotropic actions and the possibility of an actual reduction in DN [97]. Also, recent large phase III data shows that linagliptin, DPP-4 inhibitor, significantly reduces albuminuria by 30% in DN [98]. However, the role of DPP-4 inhibitors in the regulation of inflammatory cytokines and vasotropic actions remains largely unexplored (Figure 1).

## 8. Summary

 Although good glycemic control may be the best prevention of DN, it develops in spite of treatment of diabetes. Inhibitors of oxidative stress and inflammation should provide useful targets for therapy; however, many clinical trials using agents directly against these targets remain controversial. Novel therapeutic agents for patients with DN beyond glucose lowering should be highly attractive. Thus, we proposed the possible new treatments that could combine PKC*β* inhibitors with higher doses of GLP-1 agonists. GLP-1 is one potential pharmaceutical that could decrease the toxic effects, oxidative stress and inflammation of Ang II to lower the risk of DN.
